# Cross-cultural adaptation of the Scale of Perception of Respect for and Maintenance of the Dignity of the Inpatient (CuPDPH) to Brazilian Portuguese and its psychometric properties—A multicenter cross-sectional study

**DOI:** 10.1016/j.clinsp.2024.100328

**Published:** 2024-02-26

**Authors:** Pablo Eduardo Pereira Dutra, Laiana Azevedo Quagliato, Filipe Terra Curupaná, Letícia Zangirolami Peres, Victoria Luiza Pacini, Claudia Regina Menezes da Silva, Juliana Seixas Garcia, Beatriz Campillo Zaragoza, Antonio Egidio Nardi

**Affiliations:** a Laboratory of Panic and Respiration (LABPR), Institute of Psychiatry (IPUB), Federal University of Rio de Janeiro (UFRJ), RJ, Brazil; b Lourenço Jorge Municipal Hospital (HMLJ), Rio de Janeiro City Hall, Rio de Janeiro, RJ, Brazil; c Escola Universitària de Infermeria Hospital de la Santa Creu i Sant Pau, Universitat Autònoma de Barcelona, Barcelona, Spain

**Keywords:** Intimacy, Identity, Respect, Consideration, Dignity, Cross-cultural adaptation

## Abstract

**Objective:**

To adapt the Scale of Perception of Respect for and Maintenance of the Dignity of the Inpatient (CuPDPH) to the Brazilian language and culture and to assess its psychometric properties.

**Results:**

The scale was evaluated by 15 experts, and 239 patients from three tertiary hospitals in Rio de Janeiro. All participants signed a consent form. Data have shown adequacy of the model (KMO=0.839, Bartlett's test of sphericity: χ^2^(171) = 2241.3, *p* = 0.000010), good adjusted content validity (CVC_a_ ≥ 0.90), internal consistency and reliability, such as *α* = 0.927.

**Discussion:**

CuPDPH is a rating scale on observable professional attitudes. Illnesses change lives and impose adaptation to a new situation, perceived as depersonalization, leading patients to try to regain control of their lives. Patients expressed “ill will” to fill out the scale. Psychiatric patients’ scale filling time was higher than others. A sample from three Rio de Janeiro third-level hospitals may not reflect the country's population; also, this adaptation may not comprise all linguistic variations of Brazilian Portuguese and Portuguese-speaking countries.

**Conclusion:**

The Portuguese version of the Scale of Perception of Respect for and Maintenance of the Dignity of the Inpatient (CuPDPH), a 19-item, six-component version is a reliable instrument to measure the perception of internal medicine, surgical, and psychiatric patients on the maintenance of their dignity in Rio de Janeiro, Brazil. This knowledge could be used in advancing research on patients’ perception of dignity, as well as professional ethical competencies, staff-patient relationship skills, and leadership development in medical and other healthcare professional education.

## Highlights

• Despite their importance, dignity and respect are woefully undefined, and often cast aside, which is reflected by the scarcity of in-depth studies.
• Brazilian patients feel respected, but when it comes to consideration and integrity, it is nothing short of heartbreaking.
• Brazilian health professionals do not ask their patients whom they would like to share information with. And this demands our utmost attention.
• A sting of disrespect and affronts to our dignity seldom escape our notice.
• The Scale of Perception of Respect for and Maintenance of the Dignity of the Inpatient sheds light on this aspect of human interaction.

## Introduction

Studies on dignity and respect were first published in the 1960s in a few countries, especially those with psychiatric patients.^1^^,^^2^ They are overlapping concepts,^3^ poorly understood by the general population,^4^ and disrespected around the world.^5^ Specifically in healthcare settings, patients consider it important to be respected and dignified.^6^

Legally, they are fundamental human rights, endorsed by the Universal Declaration of Human Rights,^7^ the Declaration on the Promotion of Patients’ Rights in Europe,^8^ and the Universal Declaration on Bioethics and Human Rights.^9^

The Scale of Perception of Respect for and Maintenance of the Dignity of the Inpatient [in Spanish, Cuestionario de Percepción de Dignidad de Paciente Hospitalizado (CuPDPH)] was developed^2^ and validaded^4^ in Spain. It contains 19 items divided into 6 dimensions (Table 1).

**Table 1:** Dimensions of the Scale of Perception of Respect for and Maintenance of the Dignity of the Inpatient (CuPDPH)

Scale of Perception of Respect for and Maintenance of the Dignity of the Inpatient (CuPDPH)			
Factor (Dimension)	Items	Scores	Equivalence of points
F1 – Intimacy	1, 2, 3, 4, 5, 6	1 to 5 (Likert scale)	1 = 0.00, 2 = 0.25, 3 = 0.50, 4 = 0.75, 5 = 1.00
F2 – Integrity	7, 8, 9		1 = 1.00, 2 = 0.75, 3 = 0.50, 4 = 0.25, 5 = 0.00
F3 – Identity	10, 11		1 = 0.00, 2 = 0.25, 3 = 0.50, 4 = 0.75, 5 = 1.00
F4 – Information	12, 13		1 = 0.00, 2 = 0.25, 3 = 0.50, 4 = 0.75, 5 = 1.00
F5 – Respect	14, 15, 16, 17		1 = 0.00, 2 = 0.25, 3 = 0.50, 4 = 0.75, 5 = 1.00
F6 - Consideration	18, 19		1 = 0.00, 2 = 0.25, 3 = 0.50, 4 = 0.75, 5 = 1.00

**CuPDPH** = Cuestionario de percepción de dignidad de paciente hospitalizado; **F** = factor. F2 is formulated negatively to avoid response bias.

Several studies highlight the need to promote dignity in hospital environments.^5^ The quality control statistics numbers may reflect quality in terms of cleanliness, adequacy of space, reduction of wait times in emergency rooms or outpatient clinics, reduction in mortality, reduction in the rate of infection, and reduction in antibiotic use, but these numbers do not reflect the perception of dignified treatment.^10^ Those statistics reflect administrative quality, but they do not reflect the quality of the healthcare staff assistance in the perception of patients.

The pressure imposed by the administration can lead professionals to violate patients’ sense of dignity, even though patients perceive it as vital for them.^11^

Cultural adaptation is a cost-effective process in terms of resources and time, rather than creating a measure from scratch, but it has distinct phases and great methodological rigor.^12^ The authors chose to follow the methodology proposed by Borsa et al. (2012).

The objective of this study is to adapt CuPDPH to the Brazilian Portuguese and culture and to determine its internal consistency.

## Materials and methods

### Translations

On 12/02/2020, the corresponding author requested authorization from Dr. Beatriz Campillo (author of the original scale) to culturally adapt the CuPDPH scale to Brazilian Portuguese.

**Fig. 1:**
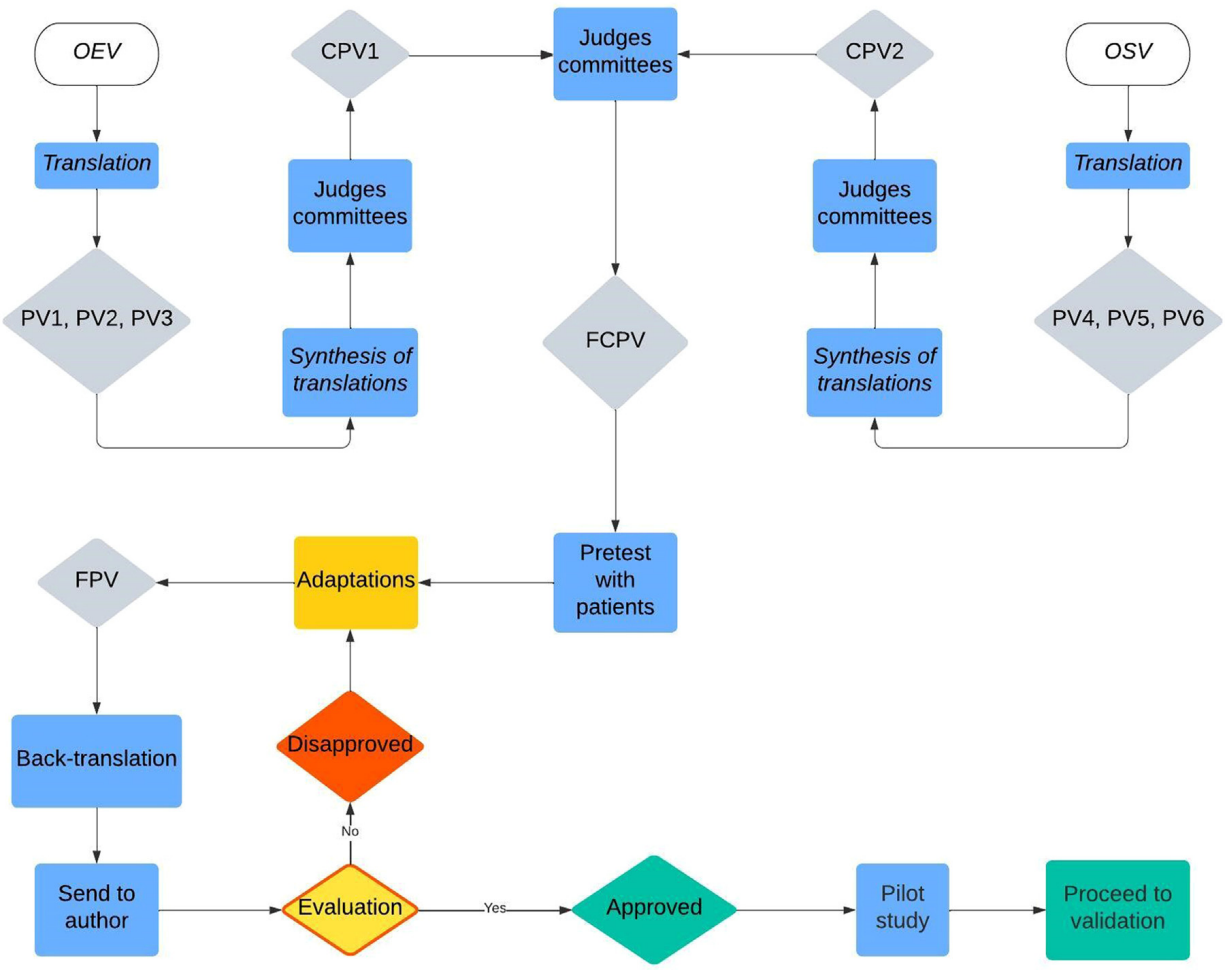
Flowchart of cultural adaptation and validation processes (OEV, Original English Version; OSV, Original Spanish Version; PV1-6, Portuguese versions 1 to 6; CPV1-2, Consensus Portuguese versions 1 and 2; FCPV, Final Consensus Portuguese Version; FPV, Final Portuguese Version).

Both the Spanish and English versions of the scale were translated to Brazilian Portuguese, each by two certified blinded translators (PV1, PV2, PV4, PV5). A third translation of each version was obtained by non-blinded translators (PV3, PV6) – (Fig. 1).

### Synthesis of translations

The six Portuguese versions (PV 1-6) were evaluated by the research group and the Committee of experts and synthesized into two Consensus Portuguese Versions (CPV1, CPV2), which were then synthesized into a Final Consensus Portuguese Version (FCPV) by consensus of more than 80% of the experts (Fig. 1 and Table 2).

**Table 2:** Original Spanish Version (OSV), Original English Version (OEV), Final Consensus Portuguese Version (FCPV).

Dimensions	Va	Original Spanish Version (OSV), Original English Version (OEV), Final Consensus Portuguese Version (FCPV)	
**F1 – Intimacy** Points equivalence: 1=0.00; 2=0.25; 3=0.50; 4=0.75; 5=1.00.	**1**	OSV	El personal me ha mirado a los ojos al hablarme They looked me in the eyes A equipe me olhava nos olhos quando falava comigo
		OEV	
		FCPV	
	**2**	OSV	He dispuesto de suficiente intimidad al usar la cuña o la botella I had privacy when using the wedge or the bottle Eu tinha privacidade suficiente para usar o banheiro ou o urinol (penico, patinho, comadre, papagaio)
		OEV	
		FCPV	
	**3**	OSV	El personal ha llamado a la puerta antes de entrar en la habitación They knocked on the door Antes de entrar no quarto/enfermaria, a equipe pedia licença ou batia na porta
		OEV	
		FCPV	
	**4**	OSV	El personal ha invitado a salir a los acompañantes del otro paciente antes de hacer algún procedimiento If I had to undergo a procedure, they asked the other patient's visitors to leave the room Quando eu precisava passar por um procedimento, a equipe pedia aos acompanhantes dos outros pacientes que saíssem do quarto/enfermaria
		OEV	
		FCPV	
	**5**	OSV	El personal tomó medidas para evitar exponer mi cuerpo innecesariamente They avoided unnecessary exposure of my body A equipe evitava expor meu corpo sem necessidade
		OEV	
		FCPV	
	**6**	OSV	He podido hablar a solas de mi situación y estado de salud, tratamiento o procedimiento con el personal I was able to discuss my situation privately with the staff Eu podia falar sobre a minha situação e estado de saúde, tratamento ou procedimento em particular com a equipe
		OEV	
		FCPV	
**F2 – Integrity** Points equivalence: 1=1.00; 2=0.75; 3=0.50; 4=0.25; 5=0.00.	**7**	OSV	El personal ha mostrado superioridad sin importarle mi opinión ni mis necessidades They showed superiority A equipe se mostrava superior (arrogante, prepotente, soberba), sem se importar com a minha opinião ou minhas necessidades
		OEV	
		FCPV	
	**8**	OSV	En ocasiones me he sentido tratado como un objeto I felt like I was treated like an objetct Em alguns momentos, senti que fui tratado como um objeto
		OEV	
		FCPV	
	**9**	OSV	El personal que me atendió hablaba como si no estuviera delante, me he sentido invisible I felt invisible A equipe que me atendia falava como se eu não estivesse no quarto/enfermaria, e eu me sentia invisível (ignorado, como se eu não existisse)
		OEV	
		FCPV	
**F3 – Identity** Points equivalence: 1=0.00; 2=0.25; 3=0.50; 4=0.75; 5=1.00.	**10**	OSV	Me han llamado por mi nombre They called me by my name A equipe me chamava pelo meu nome
		OEV	
		FCPV	
	**11**	OSV	Siento que he sido tratado con respeto sin tener en cuenta mi condición (edad, nivel cultural, o país de origen...) I did not feel discriminated against Eu sinto que fui tratado com respeito, eu não me senti discriminado (por cor, raça, idade, sexo, sexualidade, religião, país de origem etc.)
		OEV	
		FCPV	
**F4 – Information** Points equivalence: 1=0.00; 2=0.25; 3=0.50; 4=0.75; 5=1.00.	**12**	OSV	He sido informado de los detalles de mi procedimiento/tratamiento/operación I was informed Eu era informado sobre os detalhes do procedimento, tratamento ou operação
		OEV	
		FCPV	
	**13**	OSV	El personal ha dado respuestas claras a mis preguntas They provided me with clear answers A equipe deu respostas claras às minhas perguntas
		OEV	
		FCPV	
**F5 – Respect** Points equivalence: 1=0.00; 2=0.25; 3=0.50; 4=0.75; 5=1.00.	**14**	OSV	El personal ha utilizado un lenguaje respetuoso sin usar apodos o formas familiares (cariño, abuelo o querido) They used respectful language [they did not call me love, honey, darling...] A equipe usava uma linguagem respeitosa (não me chamavam por apelidos, xingamentos ou palavrões)
		OEV	
		FCPV	
	**15**	OSV	El personal ha procurado mantener mi imagen corporal (me han cubierto si llevaba bata abierta) They preserved my image A equipe procurava manter minha imagem corporal (me cobria quando era necessário)
		OEV	
		FCPV	
	**16**	OSV	He sentido que mis derechos estaban protegidos con el personal que me trató I felt my rights were protected Senti que meus direitos foram respeitados pela equipe que fez meu tratamento
		OEV	
		FCPV	
	**17**	OSV	El personal me ha dedicado el tiempo necesario para mi atención They took the time to assist me A equipe dedicava o tempo necessário para o meu tratamento/meu cuidado
		OEV	
		FCPV	
**F6 – Consideration** Points equivalence: 1=0.00; 2=0.25; 3=0.50; 4=0.75; 5=1.00.	**18**	OSV	Si en algún momento he estado preocupado o he tenido miedos relacionados con mi enfermedad o tratamiento, los profesionales me han ofrecido la oportunidad de hablar de ello They allowed me to express my feelings and worries Se em algum momento eu estive preocupado ou tive receios relacionados à minha doença ou tratamento, a equipe me permitia falar sobre isso
		OEV	
		FCPV	
	**19**	OSV	El personal me ha preguntado con quién quería compartir la información sobre mi enfermedad. They asked me who I wanted to share information with A equipe me perguntava com quem eu gostaria de compartilhar informações sobre minha doença
		OEV	
		FCPV	

F1: Intimacy; F2: Integrity; F3: Identity; F4: Information; F5: Respect; F6: Consideration; Va: Variable; OSV = Original Spanish Version; OEV = Original English Version; FCPV: Final Consensus Portuguese Version.

### Design and participants

A multicenter cross-sectional study was conceptualized to adapt CuPDPH to Brazilian Portuguese. The project began during the new Coronavirus Disease 2019 (COVID-19) pandemic, so professionals were contacted by email in the second quarter of 2021.

Experts were eligible if they agreed to participate, were fluent in English or Spanish, and were familiar with psychometrics. Those who refused to participate were excluded. The English Committee members were six psychiatrists from Federal University of Rio de Janeiro (UFRJ) (one with a postdoctoral degree, three with a doctorate degree, and two with a master's degree), two psychologists (one postdoc degree, one master's degree) and two nurses (one postdoctoral degree, one master's degree). The Spanish Committee members were three psychologists (master's degree) and two psychiatrists (one doctor degree, one master's degree).

Sample size calculation for the pretest followed the criteria of data saturation: the corresponding author interviewed patients individually until the point when no new suggestions for item modifications arose from three consecutive interviews.^13^ The authors asked them to evaluate the clarity, adequacy, and comprehension of the items, and whether any changes would be necessary to make the items more understandable.

The pilot study sample size was calculated to be within the range of 5‒10 participants for items of the scale^14^^,^^15^ and not less than 100. Therefore, the authors decided to collect 10 per item. Considering a maximum loss/dropout of 20%, the sample size would range from a minimum of 190–237 participants.

Patients’ eligibility criteria were the length of stay (>1 day), age (>18 years), and agreement to participate in the study (signed informed consent). Conversely, the exclusion criteria were being <18 years old, not signing the informed consent, being illiterate, or with any condition that could affect communication or consciousness (such as delirium).

### Data collection

Data collection was carried out by the corresponding author and by trained members of the research team at three tertiary hospitals in Rio de Janeiro: Clementino Fraga Filho University Hospital (HUCFF), Institute of Psychiatry at UFRJ (IPUB/UFRJ) and Lourenço Jorge Municipal Hospital (HMLJ).

Data from experts were collected between November 2021 and December 2021: age, sex, scale filling time, specialty, degree, and length of work experience. The authors asked them to evaluate the clarity, pertinence, and relevance of each item.

Convenient samples of patients were selected for both pretest and pilot study^14^^,^^15^ from September 2022 to February 2023: age, sex, scale filling time, hospital (HUCFF, IPUB, HMLJ), ward (internal medicine, surgical, or psychiatric), length of stay, and level of education.

### Data analysis

Participants' data were entered into a Microsoft Excel spreadsheet, version 16.70 for MacOSX to calculate the Content Validity Coefficient (CVC) and the adjusted CVC (CVC_a_).^16^^,^^17^ The cutoff was 0.80.

Sociodemographic data were described by frequencies and percentages or means and Standard Deviation (SD) and analyzed using IBM SPSS Statistics software® (IBM Corporation, NY, USA),^18^ version 29.0.0.0 for MacOSX, depending on their nature. To compare groups, the χ^2^ test, Fisher's exact test, and Student's T test was used, and the normality distribution of continuous variables was evaluated using the Kolmogorov–Smirnov and Shapiro–Wilk tests. To compare the groups in relation to age, length of stay, and time to fill the scale, a one-way analysis of variance (ANOVA-One Way) was performed. Data normality was assessed using the Kolmogorov–Smirnov and Shapiro–Wilk tests, and the assumption of homogeneity of variance was assessed using Levene's test. Once the abnormality in the distribution of continuous data was verified, bootstrapping procedures were carried out (1000 re-samplings; 95% IC BCa) to obtain greater reliability of the results and correct deviations from the normality of the sample distribution and differences between groups.^18^^,^^19^ Considering the heterogeneity of variance, Welch correction and *post-hoc* evaluation using the Games-Howell technique were requested.^18^

According to Borsa *et al*. (2012) a pilot study is the last step of cultural adaptation. The authors ran a Kaiser–Mayer–Olkin Test (KMO) to assess the appropriateness of using factor analysis on the dataset; a Bartlett's Test of Sphericity (BTS) to test the null hypothesis that the variables in the population correlation matrix are uncorrelated; and Cronbach's alpha, McDonald's Omega, and goodness of fit indices analysis to evaluate the reliability, and internal consistency of the model for this sample. The authors ran an Exploratory Factor Analysis (EFA) to check if the model obtained in Brazil would fit the Spanish one. For EFA the authors employed Factor Analysis software^20^ version 12.03.02 for Windows 64-bit, with the following parameters: method of extraction was maximum likelihood, oblimin rotation with a fixed number of factors (*n* = 6); assumption checks were Bartlett's Test of Sphericity and KMO for sampling adequacy; factor loadings <0.30 hidden and inter-factor correlations in the additional output.

### Ethical approval

This study was registered and approved by the Research Ethics Committees of the Institute of Psychiatry of the Federal University of Rio de Janeiro (IPUB/UFRJ) (CAAE 44236621.9.0000.5263, approval: 4.678.189), of the Clementino Fraga Filho University Hospital of the Federal University of Rio de Janeiro (HUCFF/UFRJ) (CAAE 44236621.9.3001.5257, approval: 5.035.181) and the Municipal Health Secretariat of the City of Rio de Janeiro (SMS-RJ), responsible for the Lourenço Jorge Municipal Hospital (HMLJ) (CAAE 33106920.5.0000.5279, approval: 5.118.710). Written informed consent was obtained from all the participants.

## Results

The author of CuPDPH consented to the cross-cultural adaptation to Brazilian Portuguese after the corresponding author e-mailed her in December 2020.

Descriptive statistics for experts, pretest, and pilot study participants are shown in Table 3. There was no statistical difference regarding age [t(13) = 0.959; *p* > 0.05], time in the profession [t(13) = 0.495; *p* > 0.05] and scale filling time [*t*(13) = 0.564; *p* > 0.05] between the English and Spanish committees of experts. However, the groups were heterogeneous in relation to gender [(χ^2^(1) = 2.727; *p* = 0.003], profession [(χ^2^(2) = 2.850; *p* = 0.004], specialty [(χ^2^(2) = 1.500; *p* = 0.004], and degree [(χ^2^(2) = 2.000; *p* = 0.003]. Among the experts’ committees the scale filling time means were higher for English (range 10‒60 min) than for Spanish committee members (range 17‒33 min).

**Table 3:** Descriptive statistics of experts, pretest, and pilot study participants.

Descriptive statistics of Experts				
		Age (years) [Mean±SD]	Scale filling time (min) [Mean±SD]	Time in the profession (years) [Mean±SD]
**Sex**	**F (n=11)**	37.00±7.80	26.18±10.62	11.18±6.19
	**M (n=4)**	39.25±15.96	35.00±19.15	14.25±16.05
**Profession**	**Medicine (n=8)**	38.75±10.54	26.62±16.01	14.62±10.20
	**Psychology (n=5)**	35.60±8.53	27.00±9.46	7.60±4.56
	**Nursing (n=2)**	38.00±16.97	40.00±0.00	12.50±14.85
**Degree**	**Master's (n=9)**	37.89±11.62	27.33±15.08	11.56±10.83
	**Doctorate (n=3)**	37.33±5.03	24.00±12.16	13.00±5.57
	**Postdoc (n=3)**	37.00±8.27	36.67±5.77	12.33±9.45
**Specialty**	**Psychiatry (n=8)**	38.75±10.54	26.62±16.01	14.62±10.20
	**CBT (n=5)**	35.60±8.53	27.00±9.46	7.60±4.56
	**Mental health (n=2)**	38.00±16.97	40.00±0.00	12.50±14.85
**Comittee**	**English (n=10)**	37.50±11.41	30.00±15.63	13.20±10.94
	**Spanish (n=5)**	37.80±7.33	25.60±6.91	9.60±3.97

**Descriptive statistics of Pretest and Pilot Study Participants**				
		**Ward**	**Pretest (n=36)**	**Pilot study (n=203)**

**Sex**	**n (F)/(M)**	Surgery	5.00/8.00	21.00/43.00
		Internal Medicine	7.00/10.00	56.00/33.00
		Psychiatry	2.00/4.00	29.00/21.00
**Age (years)**		Surgery	51.62±13.56	47.38±16.66
		Internal Medicine	56.18±16.21	48.52±17.70
		Psychiatry	29.67±8.21	35.14±10.05
**Length of stay (days)**		Surgery	11.62±5.82	17.09±16.88
		Internal Medicine	11.06±10.15	18.16±16.56
		Psychiatry	94.00±102.51	22.84±19.98
**Scale filling time (min)**		Surgery	14.08±6.73	16.50±6.83
		Internal Medicine	16.06±7.37	15.92±6.59
		Psychiatry	23.33±6.83	18.44±6.36

**n =** number of participants; **min** = minutes; **F** = female; **M** = male; **SD** = standard deviation; **CBT** = cognitive-behavioral therapy.

Among pretest patients, the groups appear to be homogeneous in relation to the variables gender [(χ^2^(2) = 0.116; *p* = 1.00] and level of education [(χ^2^(4) = 7.507; *p* = 0.185], and showed significant differences regarding age [F(2.35) = 7.734; *p* < 0.05), length of stay [F(2.35) = 10.335; *p* < 0.05), and scale filling time [F(2.35) = 3.607; *p* < 0.05). The scale filling time was higher for psychiatric participants (range 12‒31 min) than for internal medicine (range 4‒27 min) and surgical (7‒28 min) ones.

Likewise, among the pilot study sample, once the abnormality in the distribution of continuous data was verified, bootstrapping procedures were carried out (1000 re-samplings; 95% IC BCa) to obtain greater reliability of the results and correct deviations from the normality of the sample distribution and differences between groups.^19^ Considering the heterogeneity of variance, Welch correction and post-hoc evaluation using the Games-Howell technique were requested.^18^ The groups appear to be heterogeneous in relation to sex [χ^2^(4) = 24.341; *p* = 0.000], and education [(χ^2^(2) = 14.416; *p* = 0.001], and showed a statistical difference only in the age variable [F(2.200) = 12.636; *p* < 0.05)].

CVCa were calculated from ratings by experts and pretest patients with a minimum of 80% agreement among them (Table 4). The minimum CVC_a_ was 0.87 and the maximum was 0.98 for experts, and 0.96 and 0.97 for pretest participants. In addition, the pretest participants' agreement on not modifying the items was 0.97.

**Table 4:** Content validity coefficient calculation from experts’ committees (clarity, pertinence, and relevance) and from patients’ committees (clarity, adequacy, comprehension, and need to change).

	Adjusted Content Validity Coefficient (CVC_a_)									
	Experts' Committee						Patients' Committee			
	Spanish version (n=5)			English version (n=10)			Patients' pretest (n=36)			
Item	Clarity	Pertinence	Relevance	Clarity	Pertinence	Relevance	Clarity	Adequacy	Comprehension	Do not change
**1**	0.96	0.80	0.88	0.90	0.90	0.90	0.97	0.97	0.99	1.00
**2**	0.92	0.96	0.84	0.90	0.90	0.84	0.97	0.94	0.94	0.94
**3**	1.00	0.96	0.92	0.86	0.84	0.82	0.94	0.98	0.99	1.00
**4**	0.96	0.88	0.84	0.88	0.86	0.86	0.94	0.98	0.96	0.92
**5**	1.00	0.96	1.00	0.90	0.88	0.88	0.93	0.97	0.94	0.92
**6**	1.00	1.00	1.00	0.88	0.88	0.82	0.97	0.99	0.99	0.97
**7**	0.92	0.92	0.88	0.84	0.84	0.84	0.94	0.97	0.97	1.00
**8**	1.00	1.00	1.00	0.90	0.90	0.90	0.94	0.95	0.96	0.97
**9**	0.96	1.00	0.92	0.86	0.86	0.88	0.91	0.93	0.94	0.92
**10**	0.96	0.92	0.88	0.90	0.90	0.90	0.98	0.99	1.00	0.97
**11**	0.96	0.96	0.96	0.90	0.90	0.86	0.99	0.99	0.99	1.00
**12**	1.00	0.92	0.92	0.86	0.86	0.86	0.98	0.98	0.98	0.94
**13**	1.00	1.00	1.00	0.90	0.90	0.90	0.96	0.95	0.96	0.97
**14**	0.96	0.96	0.88	0.88	0.88	0.90	0.98	0.99	0.99	1.00
**15**	1.00	1.00	0.96	0.90	0.90	0.88	0.97	0.99	0.94	0.92
**16**	1.00	1.00	1.00	0.88	0.86	0.88	0.96	0.97	0.98	1.00
**17**	0.96	0.96	0.92	0.86	0.86	0.90	0.98	0.99	0.98	1.00
**18**	1.00	1.00	1.00	0.88	0.88	0.84	0.97	0.98	0.99	1.00
**19**	1.00	1.00	0.96	0.90	0.88	0.86	0.94	0.97	0.96	0.97
**CVC_a_**	**0.98**	**0.96**	**0.94**	**0.88**	**0.88**	**0.87**	**0.96**	**0.97**	**0.97**	**0.97**

CVC_a_ = Adjusted Content Validity Coefficient; Clarity, Pertinence, and Relevance (Cutoff >0.80); Clarity, Adequacy, and Comprehension (Cutoff >0.80), Do not change = Does not need to be changed (Cutoff >0.90).

The Final Consensus Portuguese Version (FCPV) was back-translated to Spanish and sent to the author of CuPDPH for evaluation. After her agreement, the research group proceeded to pilot the study. The authors expected to collect data from 190 to 237 participants, so we stopped at *n* = 203.

Pilot study participants’ rating scores mean and standard deviation (SD) for each item of the scale (variables V1‒V19) and for its dimensions (factors F1–F6) are shown in Table 5: item V14 (“they used respectful language…”) has the highest scores mean, and item V19 (“They asked me who I wanted to share information with”) the lowest one. Among factors/dimensions, the lowest and highest scores are F2 (integrity) and F3 (information), respectively. The authors also found a negative reaction from patients when asked to read the instructions and fill out the scale.

**Table 5:** Pilot study scores means for items (variables) and dimenstions (factors).

Pilot study scores means for variables/items and factors/dimensions															
V/F	Mean±SD	S	n	V/F	Mean±SD	S	n	V/F	Mean±SD	S	n	V/F	Mean±SD	S	n
**V1**	3.19±1.17	1	18	**V6**	3.20±1.16	1	17	**V11**	3.29±1.16	1	19	**V16**	3.24±1.08	1	11
		2	44			2	39			2	34			2	46
		3	46			3	62			3	44			3	51
		4	71			4	56			4	82			4	74
		5	24			5	29			5	24			5	21
**V2**	2.98±1.22	1	28	**V7**	2.74±1.27	1	33	**V12**	3.28±1.11	1	13	**V17**	3.28±1.22	1	15
		2	49			2	69			2	40			2	46
		3	47			3	45			3	52			3	51
		4	58			4	29			4	73			4	74
		5	21			5	27			5	25			5	21
**V3**	2.81±1.19	1	34	**V8**	2.73±1.29	1	33	**V13**	3.13±1.09	1	12	**V18**	2.85±1.22	1	14
		2	48			2	74			2	52			2	94
		3	60			3	41			3	57			3	35
		4	45			4	25			4	61			4	28
		5	16			5	30			5	21			5	32
**V4**	3.04±1.14	1	21	**V9**	2.82±1.31	1	32	**V14**	3.38±1.10	1	11	**V19**	2.62±1.22	1	32
		2	47			2	68			2	35			2	88
		3	55			3	38			3	52			3	37
		4	63			4	35			4	75			4	18
		5	17			5	30			5	30			5	28
**V5**	3.37±1.22	1	15	**V10**	3.27±1.21	1	11	**V15**	3.16±1.12	1	13	**Scores means for each fator (mean±SD)**			
		2	39			2	58			2	49	**F1**	3.09±0.15	**F4**	3.21±0.11
		3	48			3	35			3	57	**F2**	2.74±0.01	**F5**	3.27±0.16
		4	58			4	63			4	60	**F3**	3.28±0.01	**F6**	2.74±0.16
		5	43			5	36			5	24				

Pilot study mean scores (variables and factors). CI = Confidence interval; V = variable; F = Factor; S = Score; n = number of patients who selected each score; SD = Standard deviation; F1 = Intimacy, F2 = Integrity, F3 = Identity, F4 = Information, F5 = Respect, F6 = Consideration.

Kaiser-Meyer-Olkin test results (KMO = 0.839) indicate the adequacy of the scores for factor analysis. Bartlett's Test of Sphericity results (χ^2^ = 2243.1, df = 171, *p* = 0.000010) rejected the null hypothesis that the variables are uncorrelated. Exploratory factor analysis (EFA) provided good indicators of internal consistency and reliability (Cronbach's α = 0.927). As shown in (Table 6), some variables of the scale were highly correlated (> 0.50), as well as some of its dimensions, although the factor loading matrix of this sample has suggested a different factor solution from the original.

**Table 6:** Pilot study exploratory factor analysis statistics.

Smoothing of Correlation Matrix																			
	V1	V2	V3	V4	V5	V6	V7	V8	V9	V10	V11	V12	V13	V14	V15	V16	V17	V18	V19
**V1**	-	.536	.524	.406	.307	.334	-.367	-.366	-.395	.480	.502	.348	.366	.550	.692	.644	.679	.655	.561
**V2**		-	.584	.517	.311	.388	-.373	-.341	-.452	.362	.388	.265	.333	.286	.418	.327	.288	.220	.212
**V3**			-	.621	.279	.273	-.587	-.556	-.592	.730	.517	.622	.714	.252	.403	.389	.468	.353	.362
**V4**				-	.445	.260	-.434	-.452	-.502	.386	.227	.281	.342	.070*	.180	.169*	.351	.312	.374
**V5**					-	.826	.181	.164*	.160*	.300	.134	.225	.165*	.059*	.129*	.175	.363	.308	.363
**V6**						-	.179*	.102*	.152*	.121*	.119*	.177*	.181*	.149*	.263	.221	.209	.151*	.181
**V7**							-	.951	.889	-.509	-.395	-.489	-.553	-.316	-.389	-.374	-.336	-.270	-.330
**V8**								-	.875	-.490	-.352	-.523	-.589	-.335	-.379	-.374	-.334	-.256	-.325
**V9**									-	-.512	-.458	-.409	-.543	-.287	-.422	-.343	-.304	-.305	-.356
**V10**										-	.771	.800	.730	.274	.369	.397	.545	.473	.406
**V11**											-	.618	.625	.387	.424	.388	.382	.313	.208
**V12**												-	.896	.452	.426	.458	.530	.379	.294
**V13**													-	.462	.452	.431	.406	.305	.292
**V14**														-	.894	.827	.573	.395	.206
**V15**															-	.855	.657	.507	.355
**V16**																-	.740	.582	.350
**V17**																	-	.706	.555
**V18**																		-	.839
**V19**																			-
															*not significant				
**Rotated Loading Matrix**									**Interfactors Correlation Matrix**										
	**F1**	**F2**	**F3**	**F4**	**F5**	**F6**	**Com**			**F1**	**F2**	**F3**	**F4**	**F5**	**F6**				

**V1**				.345	.522		.715		**F1**	-									
**V2**						.615	.505		**F2**	-.564	-								
**V3**		.507				.432	.733		**F3**	.565	-.107	-							
**V4**						.657	.573		**F4**	-.339	.448	-.070	-						
**V5**			.683			.933	.877		**F5**	-.363	.523	.004	.413	-					
**V6**			.621			.896	.686		**F6**	-.402	.381	-.449	.430	.217	-				
**V7**	.932						.906												
**V8**	.950						.827		**Adequacy of the Polychoric Correlation Matrix and Robust Goodness of Fit**										
**V9**	.807						.933		BTS = 2243.1 (df=171; p=.000010)										
**V10**		.875					.804		KMO = .83957 (BCa 95% CI = .783 to .879)										
**V11**		.727					.546		α = .927226										
**V12**		.941					.881		ω = .9250028										
**V13**		.847					.837		RMSEA = .0000 (BCa 95% CI = .0000 to .1834)										
**V14**					.885		.780		CFI = .999 (BCa 95% CI = .890 to 1.003)										
**V15**					.934		.880		TLI = 1.030 (BCa 95% CI = 1.002 to 1.103)										
**V16**					.873		.883												
**V17**				.502	.441		.724												
**V18**				.906			.861												
**V19**				.817			.707												

*Correlation matrix, significantly different from zero at population (p<0.05);****EFA****= Exploratory Factor Analysis;****CI****= Confidence interval;****BCa****= Bias-corrected and accelerated;****V****= variable;****F****= Factor;****CFI****= Comparative Fit Index;****TLI****= Tucker & Lewis Index;****KMO****= Kaiser-Meyer-Olkin Test;****BTS****= Bartlett's Test of Sphericity;****RMSEA****= Root Mean Square Error of Approximation;****Com****= Communality;****F1****= Intimacy,****F2****= Integrity,****F3****= Identity,****F4****= Information,****F5****= Respect,****F6****= Consideration.*

## Discussion

CuPDPH is a 19-statement rating scale on observable professional attitudes. On the construction of CuPDPH the authors hypothesized that dignity would be the sum and inter-relation of perceivable attitudes of healthcare staff during hospitalization.^2^^,^^4^ Illnesses change lives and impose adaptation to a new situation in which patients cannot even decide when or what to eat. This is perceived as depersonalization^21^ and leads them to try to regain control of their lives. They perceive a maintenance of their dignity where their autonomy is preserved.^22^^,^^23^ Likewise, health professionals must be aware of those circumstances to provide information about what is going to happen and what is scheduled for the day. By doing so, patients feel their autonomy and dignity is being respected. If we want to increase patients’ autonomy, we must encourage them to make decisions on their own health under professional guidance. This is the only way they would feel in charge of their own choices.

CVC_a_ results and the agreement of pretest patients on not to make any further modifications of the items indicated good content validity and adaptation to Brazilian culture. Item V3 had to be adapted to certain circumstances because the original item was about knocking on the door before entering a patient's room. At the three hospitals, some patients have mentioned the lack of doors in their rooms. This is one of the suggestions that arose when interviewing pretest patients for data saturation. The solution found was to insert the semantic equivalent expression “asking permission to enter the room”. Neither the committees of experts nor the research group had thought about it previously. And this is an example of how important the pretest phase is on the cultural adaptation of the scale. Item V14 (“They used respectful language…”) had to be modified because in Brazilian culture patients do not feel offended to be called “grandmother” or “grandfather”; instead, they feel it if professionals uses swear words or insults. The authors also know that it is not adequate to call someone “grandma” or “grandpa”, but in everyday language, it was considered acceptable by this sample. Although the authors know that is always advisable to call patients by their names.

The analysis of inter-factor relations (Table 6) allows us to reaffirm the structure found on the original scale with a high correlation between the dimensions of the scale. The lowest scores mean (Table 5) was that for item V19. Likewise, the dimension Consideration (F6), to which V19 belongs, was the one with the lowest scores mean. This result is like what was found in Spain by Campillo (2020). Although confidentiality of medical information is defined by law, in some situations medical staff must breach confidentiality. Imagine an unaccompanied patient with cognitive impairment admitted to the emergency department for surgery, or unconscious due to brain hemorrhage, who could not determine a legal representative previously: although not determined by law, medical staff has an obligation to inform relatives of patients’ conditions. But with conscious patients, who can make decisions on their own, it is advisable to ask for patients’ consent before sharing any information with their relatives.

One of the experts from the English committee took 60 min to complete the scale and this may be one of the reasons why this committee’s mean time was higher than that of the Spanish committee. In addition, experts justified that they had to think and analyze many aspects of each item, and that took longer than simply fulfilling the scale. Among participants of the pretest and pilot study, the scale completion time was much higher than that found in Spain,^2^ and this may be due to differences concerning the level of education in Spain compared to Brazil.^24^ Being better educated leads to better skills in reading and text comprehension, therefore Spanish patients would complete the scale faster.

We also found a certain “ill will” when asking patients to fill the scale, and in many situations, patients asked us to read it for them instead of asking them to. This may be due to patients’ malaise, but in general, it was a certain laziness in reading it in full. The authors were not able to determine whether this was because of the low level of education, and consequently poorer reading and comprehension skills. In addition, the authors have found another barrier to be transposed by psychiatric patients, besides the low level of education. Despite being younger, their scale completion time was higher than other patients. This may be explained by their own condition because mental illnesses lead to cognitive decline.[25], [26], [27]

EFA has shown a different factor solution from the original CuPDPH. Item V1 loading for this sample was different: this may be because looking in the eyes, in Brazilian culture is not only an act of intimacy, but also an act of respect and consideration. This may be the reason why it loaded on factors F5 (Respect) and F6 (Consideration). In Brazilian culture people like to be looked in the eyes when they are in a conversation.

CuPDPH is a good instrument for addressing the quality of care from the perspective of patients, not from the administrative point of view. It addresses several aspects of patients’ perception of their intimacy, integrity, identity, information, respect, and consideration, and how they perceive them on their interaction with healthcare staff. From the educational point of view CuPDPH is supposed to be an instrument for professional skills training, and from the ethical perspective, it is an opportunity for healthcare staff and administration staff to get in line to bioethical principles. For instance, administration quality evaluation is restricted to services and supplies. Improving the respect for patients’ dignity can reduce complaints and even prevent lawsuits for professionals and hospitals.

Health service managers assess the quality of care provided based on numerical indicators produced by themselves (such as mortality rates and nosocomial infection rates). However, there is still a shortage of instruments that assess the quality of care from the perspective of the patients (stakeholders). CuPDPH fills this gap in the evaluation of the quality of care from the patient's point of view. There is a need to expand this field of study and allow for progress in this area so that patients can effectively be given the autonomy to say what is good or not and suggest improvements.

The present study is part of a master's thesis project, whose objective is to culturally adapt and validate the CuPDPH scale into Brazilian Portuguese. This article comprises the cultural adaptation stage. The authors will proceed with the process of validating the instrument for use throughout Brazil. Its psychometric properties have shown great reliability in measuring the perception of respect and dignity in the analyzed sample.

Data from this stage bring greater robustness to the study and definitively launches this scale as an important ally in the training and improvement of healthcare staff professional skills.

The study took place in three public tertiary level hospitals in Rio de Janeiro: two Federal University Hospitals (IPUB and HUCFF) and one Municipal Hospital (HMLJ), and this may be the reason why the scores were lower than what was found by the author in Spain^7^. Public Brazilian health professionals are dissatisfied due to low pay, work overload, and unhealthy working conditions, which may have an impact on their attitudes towards patients. Besides, some hospital rooms have no doors at all. Altogether, this may impact patients’ perception of dignified and respectful care.^5^

The authors also found limitations to this study. A cross-sectional study has low accuracy over time because it cannot detect future changes. The convenience sampling method can be biased^28^ because the selected participants may be prone to questioning, or complaining, which may not reflect most of the population. On the other hand, as the Brazilian health system limits access to hospital beds, patients may feel so relieved to be at one that they would not complain, and this could affect their perception of health professional attitudes. This may limit the capacity to generalize the results. The study took place in three third-level hospitals, so its results may not reflect the perception of patients from primary- and second-level care. Rio de Janeiro's population has its own peculiarities, with people living under violence and poverty roof, with difficulty to assess health system facilities. A sample from Rio de Janeiro tertiary hospitals could neither reflect most of the population of patients, nor the state or the country population. Although the authors have tried to include as many linguistic variations as possible, this study took place in southeastern Brazil, so this scale may not comprise all linguistic variations of Brazilian Portuguese and Portuguese-speaking countries.

## Conclusion

The Portuguese version of the Scale of Perception of Respect for and Maintenance of the Dignity of the Inpatient [CuPDPH], a 19-item, six-component version is a reliable instrument to measure the perception of internal medicine, surgical, and psychiatric patients on the maintenance of their dignity in Rio de Janeiro, Brazil. Validation studies in Brazil are ongoing by the research group in Rio, but further studies in other Brazilian states, as well as other Portuguese-speaking countries, will be necessary to provide evidence-based knowledge of patients’ perception of dignity.

This knowledge could be used in advancing research on patients’ perception of dignity, as well as professional ethical competencies, staff-patient relationship skills, and leadership development in medical and other healthcare professional education.

In Brazil, the instrument will be called "Escala de Avaliação da Percepção de Respeito e Manutenção da Dignidade do Paciente Internado (APREMDI)". The authors thought this anagram is better for memorization because of its similarity to the word “aprendi”, the past tense of “to learn”, in Portuguese.

## Animal involvement

No animals were involved in this research.

## CRediT authorship contribution statement

**Pablo Eduardo Pereira Dutra:** Conceptualization, Methodology, Formal analysis, Writing – review & editing. **Laiana Azevedo Quagliato:** Conceptualization, Methodology, Formal analysis, Writing – review & editing. **Filipe Terra Curupaná:** Methodology, Formal analysis. **Letícia Zangirolami Peres:** Methodology, Formal analysis. **Victoria Luiza Pacini:** Methodology, Formal analysis. **Claudia Regina Menezes da Silva:** Methodology, Formal analysis. **Juliana Seixas Garcia:** Methodology, Formal analysis. **Beatriz Campillo Zaragoza:** Formal analysis, Writing – original draft. **Antonio Egidio Nardi:** Conceptualization, Methodology, Formal analysis, Writing – review & editing.

## Declaration of competing interest

The authors declare no conflicts of interest.
